# Lead Nitrate Induces Inflammation and Apoptosis in Rat Lungs Through the Activation of NF-κB and AhR Signaling Pathways

**DOI:** 10.1007/s11356-022-19980-8

**Published:** 2022-04-28

**Authors:** Ibraheem M. Attafi, Saleh A. Bakheet, Sheikh F. Ahmad, Osamah M. Belali, Fawaz E. Alanazi, Suliman A. Aljarboa, Ibrahim A. AL-Alallah, Hesham M. Korashy

**Affiliations:** 1 Department of Pharmacology & Toxicology, College of Pharmacy, King Saud University, Riyadh, Saudi Arabia; 2 Poison Control and Medical Forensic Chemistry Center, Jazan Health Affairs, Jazan, Saudi Arabia; 3 Aseer Central Hospital, Asser health affairs, Ministry of Health, Abha, Saudi Arabia; 4 Security Forces Hospital Program, Riyadh, Saudi Arabia; 5 Central Laboratory, Research Center, College of Pharmacy, King Saud University, Riyadh, Saudi Arabia; 6 Pathology and Clinical Laboratories Medicine, King Fahad Medical City, Riyadh, Saudi Arabia; 7 Department of Pharmaceutical Sciences, College of Pharmacy, QU Health, Qatar University, Doha, Qatar

**Keywords:** Lead, Inflammation, Apoptosis, NF-κB, AhR, In vivo rat, Lung

## Abstract

Lead (Pb) is one of the most frequent hazardous air contaminants, where the lungs are particularly vulnerable to its toxicity. However, the Pb distribution and its impact on lung inflammation/apoptosis and particularly the involvement of nuclear factor kappa B (NF-κB) and aryl hydrocarbon receptor (AhR) signaling pathways in Pb-induced lung toxicity have not yet been fully investigated. Adult male Wistar albino rats were exposed to Pb nitrate 25, 50, and 100 mg/kg b.w. orally for 3 days. The histopathological changes of several rat organs were analyzed using hematoxylin and eosin staining. The concentrations of Pb ion in different organ tissues were quantified using inductive coupled plasma mass spectrometry, while gas chromatography-mass spectrometry was used to identify organic compounds. The changes in the mRNA and protein expression levels of inflammatory and apoptotic genes in response to Pb exposure were quantified by using RT-PCR and Western blot analyses, respectively. Treatment of rats with Pb for three consecutive days significantly increased the accumulation of Pb in lung tissues causing severe interstitial inflammation. Pb treatment also increased the percentage of lung apoptotic cells and modulated apoptotic genes (Bc2, p53, and TGF-α), inflammatory markers (IL-4, IL-10, TNF-α), and oxidative stress biomarkers (iNOS, CYP1A1, EphX) in rat lung tissues. These effects were associated with a significant increase in organic compounds, such as 3-nitrotyrosine and myeloperoxidase, and some inorganic elements, such as selenium. Importantly, the Pb-induced lung inflammation and apoptosis were associated with a proportional increase in the expression of NF-κB and AhR mRNAs and proteins. These findings clearly show that Pb induces severe inflammation and apoptosis in rat lungs and suggest that NF-κB and AhR may play a role in Pb-induced lung toxicity.

## Introduction

Heavy metals are the most significant air pollutants that are neither created nor biodegradable, making their exposure an increasing issue (Jarup 2003). Among these heavy metals, lead (Pb) is ranked the second most commonly encountered toxic substance according to the Agency for Toxic Substances and Disease Registry 2019 (ATSDR 2019). Human and experimental animal studies have shown that exposure to Pb affects the function of a variety of immune cells and the production of inflammatory mediators and cytokines, such as interleukins, transforming growth factor-beta1 (TGF-β1), and tumor necrosis factor-alpha (TNF-α) (Chibowska et al. 2016; Lassiter et al. 2015). These cytokines and immunomodulatory markers are regulated by several transcription factors, including the nuclear factor kappa B (NF-κB) and the aryl hydrocarbon receptor (AhR) (Beamer and Shepherd 2013; Li and Verma 2002), which both have an essential role in organ inflammation and apoptosis (Li and Verma 2002; Villa et al. 2017). Among these organs, the lungs are considered as a primary soft organ for Pb exposure and accumulation as they consist of diverse cells (Kumar et al. 1991).

AhR activation with subsequent induction of its target gene cytochrome P4501A1 (CYP1A1) has been shown to play a role in lung toxicity. Several previous studies have linked the exposure to AhR activating environmental pollutants such as 2,3,7,8-tetrachlorodibenzo-p-dioxin (TCDD) with lung inflammation and apoptosis in pheochromocytoma PC12 cells (Beamer and Shepherd 2013; Sánchez-Martín et al. 2010). This effect was associated with modulation of the immune system cells and hematopoietic stem cell differentiation (Beamer and Shepherd 2013), while was diminished in AhR-null (AhR−/−) mice or by using AhR chemical inhibitors in cerebellar granule cells (AhR+/+) cultures (Sanchez-Martin et al. 2011). On the other hand, the NF-κB family of transcription factors are ubiquitously expressed regulators of cell proliferation, oxidative stress, apoptosis, inflammation, and fibrosis of the lung (Ghosh et al. 1998). In this context, it has been shown that suppression of lipopolysaccharide-induced inflammation and oxidative stress by punicalagin was mediated through blockage of NF-κB activation (Jha and Das 2017), whereas activation of NF-κB in airway epithelium cells increased lung inflammation in mice (Sheller et al. 2009).

A cross-talk between NF-κB and AhR in lung inflammation has been reported in both in vitro and in vivo studies. In which, activation of AhR by TCDD targets NF-κB in a ligand-dependent manner (Kimura et al. 2009; Vogel et al. 2014; Vogel and Matsumura 2009), leading to the regulation of inflammatory responsive genes, such as interleukin-1 (IL-1), IL-6, and IL-8 in non-small cell lung cancer patient (Chen et al. 2012; Kobayashi et al. 2008). Interestingly, the overexpression of AhR significantly increased NF-κB activity and thus promoting the development of lung adenocarcinomas (Chen et al. 2012). However, the role and involvement of AhR and NF-κB in Pb-induced lung toxicities in the rat lung model remain uninvestigated. Therefore, the current study was conducted a) to investigate the distribution of Pb in different vital organs and tissues, b) to determine the effect on serum biomarkers, apoptosis, inflammation, the profile of organic ions, and inorganic compounds in lung tissue, and c) to explore the role of the NF-κB and AhR pathways in the Pb-induced lung damage in vivo rat model.

## Materials and methods

### Materials

Lead (II) nitrate (purity <99.95%) was purchased from Sigma-Aldrich, St. (Louis, MO, USA). Muse Annexin V and Dead Cell assay kit was obtained from Merck Millipore (Darmstadt, Germany). TRIzol reagent was obtained from Invitrogen Co., (Island, NY, USA). High-capacity cDNA Reverse Transcription and SYBR Green PCR Master Mix kits were ordered from Applied Biosystems (Foster City, CA, USA). Primary antibodies against target proteins NF-κB p65 and AhR and their HRP-conjugated secondary antibodies were purchased from Santa Cruz Biotechnology, (Santa Cruz, CA, USA). Enhanced chemiluminescence Western blot detection kit was obtained from EMD Millipore Co., (Billerica, MA, USA).

### Animal study design and treatment protocols

Wistar albino rats (adult male; 200–230 g body weight) were obtained from the Animal Care Center, King Saud University, Riyadh, Saudi Arabia. Under regulated conditions (25°C and a 12-h light/dark cycle), all animals were kept in metabolic cages and had unlimited access to a pulverized standard rat pellet diet. All animals were allowed to acclimatize to the environment in the animal facility for a week before starting the experiments. These animals were cared for and handled in accordance with Animal Care Center regulations and international guidelines (e.g., NIH 1976). The study protocol was approved by the Research Ethics Committee of King Saud University in Riyadh, Saudi Arabia (ethical approval # KSU-SE-20-33).

A total of 24 rats, a sample size that was calculated by the Resource equation method (Charan and Kantharia 2013) to give a significance of 5% and a power of 95%, were divided randomly but equally into 4 groups. The first group (control) received a single dosage of normal saline (2.5 ml/kg/day) by gavage for three consecutive days. The second (Pb 25), third (Pb 50), and fourth (Pb 100) groups received lead nitrate [Pb (NO_3_)_2_, Pb] 25, 50, and 100 mg/kg body weight (b.w.) by gavage, respectively, for three consecutive days. These doses of Pb were chosen from our previous published work (Ansari et al. 2013), which are about 1.1%, 2.2%, and 4.4%, respectively, of the reported LD_50_ (2250 mg/kg) of Pb nitrate in rats (Sharma et al. 2010), and none of the rats died during the experimental period. Animals were anesthetized with halothane at the end of the treatment period and blood samples were taken through heart puncture. The serum was separated by centrifugation at 3000×*g* for 5 min and kept at −20°C until analysis. Under halothane anesthesia, the thorax was opened, and lung, heart, and liver tissues were dissected quickly, lavaged with phosphate-buffered saline (PBS) (Collins et al. 2016), and then stored at −80°C for further analysis. Each dissected organ was divided into four segments; the first segment was homogenized and used to isolate RNAs and proteins for gene expression experiments, the second segment was fixed in 10% formalin for use in a histopathology study, the third segment was used for flow cytometry analysis, and the fourth segment was used for the analysis of inorganic ions and organic compounds (Collins et al. 2016).

### Pb distribution in rat organ tissues

The distribution of Pb in different rat organ tissues was quantified by using inductively coupled plasma mass spectrometry (ICP-MS). Using microwave digestion, all tissue samples were digested in nitric acid and the distribution of Pb in these tissues was assessed as previously described (Orct et al. 2017). Approximately 500 mg tissue was placed in a plastic bag and homogenized with sterile deionized water for 60 s by mechanical dissociation (Stomacher^®^80, lab system, France). The homogenate was transferred into a 15-mL plastic tube and centrifuged for 10 min at 3000×*g*. Supernatant from each homogenate was stored at −20°C for ICP-MS analysis. The homogenate samples were then digested in pure 70% nitric acid at 70°C for 12 h, and then dilated by ultrapure deionized water and the elemental profile was analyzed using an ICP-MS instrument (Agilent Technologies, ICPMS-7500 System) (Albratty et al. 2017).

### Serum analysis of immunological markers

The serum levels of immunological markers, including myeloperoxidase (MPO) (cat. no. 704655.10) and proteinase 3 (PR-3) (Cat. no. 704660.10) were determined by enzyme-linked immunosorbent assay (ELISA) (QUANTA Lite assay, INOVA Diagnostics, USA). Immunoglobulins (IgG, IgE, and IgA) were determined by nephelometry (Siemens BN2 nephelometer, Germany). Lactate dehydrogenase (LDH) and creatine kinase-MB (CK-MB) were determined by chemiluminescent microparticle immunoassay (CMIA) (Architect c16000 analyzer, Abbott Diagnostics Inc, USA).

### Histopathology examination

The effect of Pb on the histology of rat organs (brain, heart, and lung tissues), obtained from control rats and rats exposed to Pb (100 mg/kg b.w.), were analyzed using hematoxylin and eosin (H&E) staining, as previously described (Afshar et al. 2008) with slight modification (Ansari et al. 2013). Briefly, parts of the organ tissues were fixed in 10% formalin and then cut into 3–4-mm-thick paraffin-embedded sections. The sections were then stained with H&E for examination of histopathological changes under light microscopy by two independent histopathologists.

### Cell apoptosis

The percentage of cells undergoing apoptosis/necrosis in response to Pb was determined according to manufacturing protocols and as described previously (Khan et al. 2012). Briefly, lung cells isolated from rat lung tissues were washed with cold PBS followed by trypsinization. The collected lung cells were centrifuged at 300×*g* for 5 min and then re-suspended in 0.5 mL PBS. Thereafter, lung cells were stained with annexin V/propidium iodide (PI), and the apoptotic and necrotic cell populations were analyzed by Flow Cytometer Muse^®^ Cell Analyzer (Merck Millipore, CA, USA).

### RNA isolation, cDNA synthesis, and RT-PCR

Total RNA from lung tissues was isolated using the TRIzol method as previously described (Rio et al. 2010). RNA quantity and quality were determined using NanoDrop 8000^®^, Thermo Scientific (USA) at an optical density 260/280 range of ~2.0. cDNA synthesis was performed and the changes in the mRNA expression levels of NF-κB, AhR, CYP1A1, IL-4, IL-6, IL-8, IL-10, TNF-α, epoxide hydrolases (EphX), TGF-α, BcL2, p53, and inducible nitric oxide synthase (iNOS) (Table 1) in response to Pb exposure were quantified by QuantStudio 6 Flex Real-Time PCR System (qRT-PCR), Applied Biosystems (Foster City, CA) using SYBR Green Master mix as described by the manufacturer. The changes in gene expression normalized to an endogenous reference gene (β-actin) were calculated using the relative gene expression method (i.e., ΔΔCT) (Livak and Schmittgen 2001).

**Table 1:** Primer sequences used for real-time polymerase chain (RT-PCR) reactions

Gene	5′→3′ forward primer	5′→3′ reverse primer	References
*NF-κB*	GGCAGCACTCCTTATCAA	GGTGTCGTCCCATCGTAG	(Korashy et al. 2016a)
*CYP1A1*	CCAAACGAGTTCCGGCCT	TGCCCAAACCAAAGAGAATGA	(Korashy et al. 2016a)
*AhR*	CTCCCTCCACAGTTGGCTTTGTTTG	GATTCTGCGCAGTGAAGCATGTCAG	(Jacob et al. 2011)
*IL-4*	TGGGTCTCAGCCCCCACCTT	TCCGTGGATACCGTTCCCGGT	(de Melo et al. 2015)
*IL-6*	CCGGAGAGGAGACTTCACAGAGGA	AGCCTCCGACTTGTGAAGTGGTATA	(de Melo et al. 2015)
*IL-8*	CATTAATATTTAACGATGTGGATGCGTTTCA	GCCTACCATCTTTAAACTGCACAAT	(Tong et al. 2008)
*TNF-a*	GTGATCGGTCCCAACAAG	AGGGTCTGGGCCATGGAA	(Korashy et al. 2016a)
*EphX2*	CACATCCAAGCCACCAAGCC	CAGGCCTCCATCCTCCAG	(Ansari et al. 2013)
*TGF-a*	TCAACAAGTGCCCAGATTCCC	GGCTTCTCTTCCTGCACCAAA	(Hu et al. 2021)
*BcL2*	CAACATCGCTCTGTGGATGAC	TGGGGCCATATAGTTCCACAA	(Saleh and El-Shorbagy 2020)
*P53*	ACAGCGTGGTGGTACCGTAT	GGAGCTGTTGCACATGTACT	(Korashy et al. 2016b)
*iNOS*	GTCACCTATCGCACCCCGAGATG	GCCACTGA CACTCCGCACAAAG	(Al-Harbi et al. 2015)
*β-actin*	CCAGATCATGTTTGAGACCTTCAA	GTGGTACGACCAGAGGCATACA	(Korashy et al. 2016a)

### Protein extraction and Western blot analysis

Total proteins concentrations isolated from lung tissues of all groups were quantified using a Direct Detect Infrared Spectrophotometer (Millipore, MA, USA), as previously described (Abrams et al. 2003). Western blot analysis was performed to determine the protein expression of NF-κB and AhR normalized to β-actin as initially described before (Korashy and El-Kadi 2004). Briefly, about 30 μg of proteins from all animal groups were separated on 10–12% sodium dodecyl sulfate–polyacrylamide gel electrophoresis (SDS-PAGE) and then electrophoretically transferred to nitrocellulose membrane. After serial washings, the membranes were incubated at 4°C with specific primary antibodies against target proteins followed by secondary antibodies at room temperature. The bands of the target proteins were visualized by C-DiGit^®^ Blot Scanner (LI-COR Biosciences, USA) and then the semi-quantified by ImageJ^®^ (Rueden et al. 2017).

### Analysis of inorganic ions and organic compounds in rat blood

Lung tissue homogenates were centrifuged for 10 min at 3000×*g* and the resultant supernatants were utilized for GC-MS and ICP-MS analyses. For GC-MS analysis, lung homogenate samples were extracted by a solid phase–extracted (SPE) method according to the manufacturer’s protocol using a Clean Screen^®^ DAU SPE cartridge. The extracted residues were reconstituted by methanol and analyzed using a non-targeted screening method via a GC-MS instrument (Agilent Technologies, GCMS-7890B System). For ICP-MS analysis, lung homogenate samples were digested in pure 70% nitric acid at 70°C for 12 h which were then dilated by ultrapure deionized water. The elemental profile was analyzed using an ICP-MS instrument (Agilent Technologies, ICPMS-7500 System) as described before (Albratty et al. 2017).

### Statistical analysis

Results are expressed as mean ± standard error of the mean (SEM). Student *t*-test or one-way analysis of variance (ANOVA) followed by a Student-Newman-Keuls test were performed using Sigma Plot (Systat Software, Inc, CA) to compare the experimental groups’ results to their corresponding control group. The statistical significance was defined at a *P*-value of <0.05.

## Results

### Distribution of Pb in rat lungs a compared to heart and brain and the effect on the histological changes

**Fig. 1:**
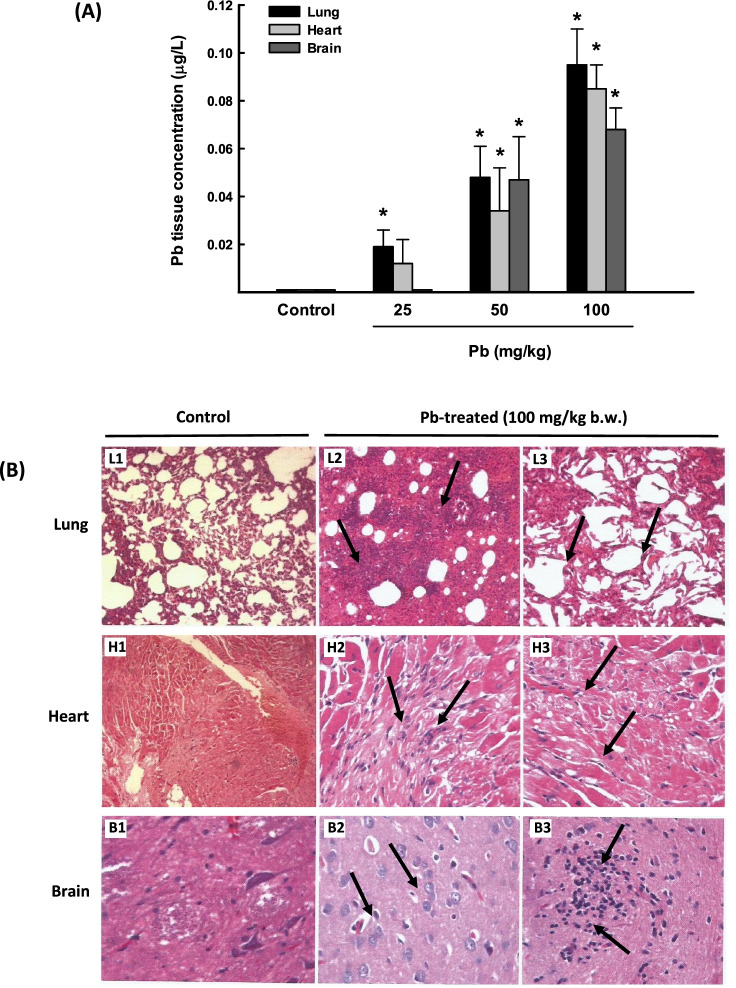
Pb distribution and histopathology changes in lung, heart, and brain tissues from rats exposed to once-daily dose of Pb for 3 days. **A** The Pb concentration levels in lung, heart, and brain tissues from rats treated with Pb (25, 50, and 100 mg/kg b.w.) were determined by ICP-MS. The values are presented as mean ± SEM (*n* = 3). *p < 0.05 compared to the control (Pb 0 mg/kg b.w.). **B** Histopathologic examination of rat lung, heart, and brain tissues from rats treated with Pb (100 mg/kg Pb b.w.) was performed using H&E staining. Representative images of lung histology of control rat (L1) show a normal architecture (50× magnification), whereas L2 (100× magnification) and L3 (200× magnification) show severe interstitial inflammation, fibrosis, and alveolar collapse in the lungs. H1 shows a normal heart (20× magnification), whereas H2 and H3 show marked congestion, interstitial inflammation, fibrosis, and focal cardiac muscle degeneration (100× magnification). B1 represents normal brain structure (200× magnification) and B2 (200× magnification) and B3 (100× magnification) demonstrate cellular degeneration and increased inflammatory cells

In the current study, we determined the distribution of Pb metal, after three days of oral administration, in rat lungs in comparison to the heart and brain tissues using the ICP/MS technique. Figure 1A shows that Pb accumulated in all tested rat tissues in a dose-dependent manner. Interestingly, the lung exhibited the highest Pb accumulation by approximately 95-fold followed by the heart by 86-fold, and 68-fold for the brain. To further explore the delirious effect of Pb and whether the high accumulation levels of Pb are associated with histopathological changes, we conducted H&E staining of the rat lung, heart, and brain tissues. Figure 1B shows that rats exposed to one single dose of Pb (100 mg/kg b.w) for 3 days exhibited severe interstitial inflammation, fibrosis, and alveolar collapse in the lungs (L2 and L3) as compared to control lung (L1). In the heart tissues, marked congestion, interstitial inflammation, fibrosis, and focal cardiac muscle degeneration were observed (H2 and H3), as compared to normal heart structure (H1). Whereas cellular degeneration and increased inflammatory cells were detected in the brain tissues (B2 and B3) as compared to normal brain (B1) structures. Based on these results, the Pb dose (100 mg/kg b.w.) was utilized in all subsequent studies in rat lung tissues.

### Effect of Pb exposure on rat lung apoptosis

**Fig. 2:**
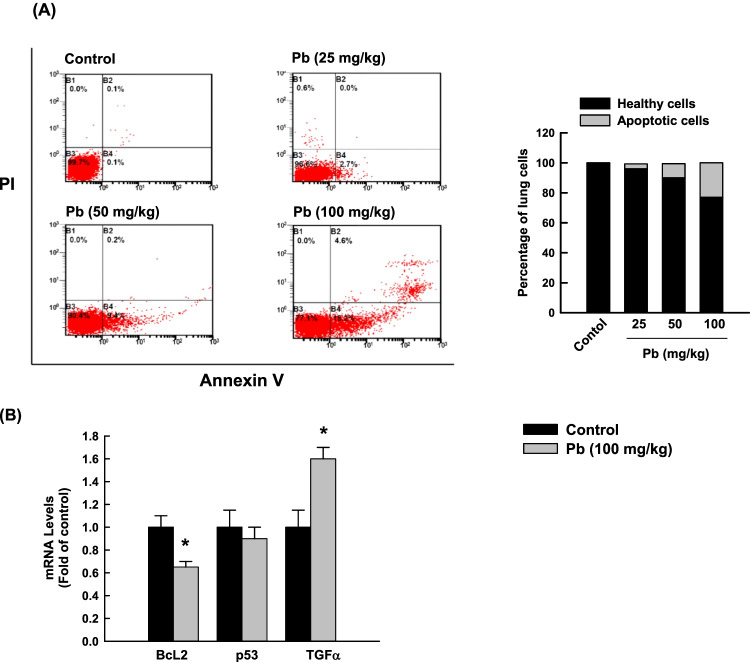
Apoptotic effects in lung cells of rats exposed to a once-daily dose of Pb for 3 days. **A** The percentage of apoptotic rat lung cells after 3 days of treatment of Pb (25, 50, and 100 mg/kg, b.w.) was determined by flow cytometry using annexin V/PI as substrates. One of the three representative experiments from different cell preparations was only shown. The values are presented as mean (*n* = 3). **B** The mRNA levels of BcL2, p53, and TGF-α normalized to the β-actin housekeeping gene were determined by RT-PCR. The values are presented as mean ± SEM (*n* = 6, duplicate). *p < 0.05 compared to the control (Pb 0 mg/kg b.w.)

The ability of Pb to induce apoptosis in rat lung cells was assessed by (1) determining the percentage of cells undergoing apoptosis/necrosis and (2) measuring the levels of BcL2, p53, and TGF-α mRNAs. Figure 2A shows that 3-day exposure to Pb (25, 50, and 100 mg/kg b.w.) significantly increased the percentage of lung cells that underwent apoptosis in a dose-dependent manner to 3.5%, 9.5%, and 22.9%, respectively, compared to control healthy cells (0.1 %). Furthermore, the Pb (100 mg/kg b.w.)-induced apoptosis in the lungs was further evidenced by the decreased expression of the anti-apoptotic gene BcL2 (by 40%), and increased expression of the pro-apoptotic TGF-α gene (by 60%) compared to control cells (Fig. 2B).

### Effect of Pb exposure on the inflammation of lung tissues

**Fig. 3:**
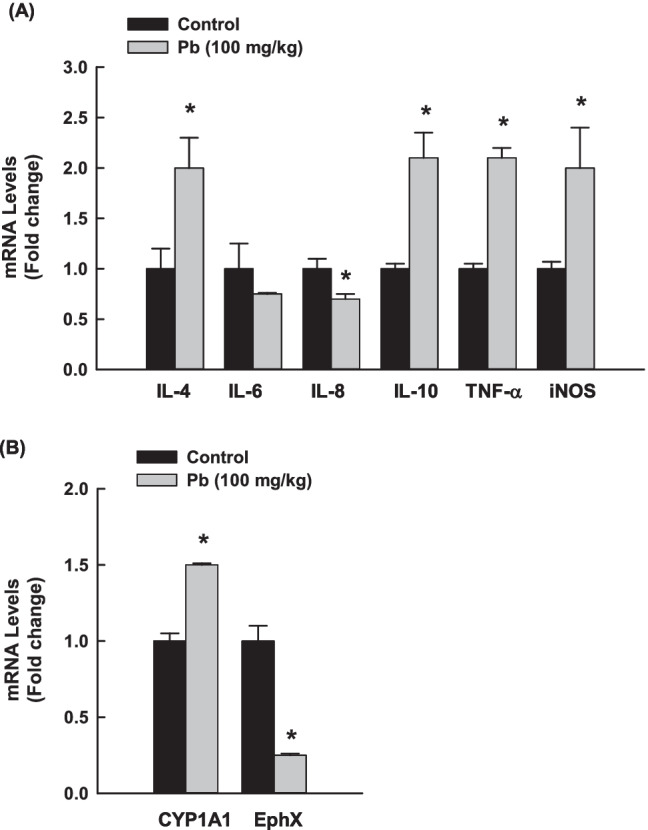
Effects of Pb on the expression of inflammatory genes in the lungs of rats exposed to once-daily Pb (100 mg/kg) for 3 days. The mRNA levels of **A** IL-4, IL-6, IL-8, IL-10, iNOS, TNF-α, and **B** CYP1A1 and EphX normalized to β-actin housekeeping gene were determined using RT-PCR. The values are presented as mean ± SEM (*n* = 6, duplicate). *p < 0.05 compared to the control (Pb 0 mg/kg b.w.)

To further understand the effect of Pb on lung inflammation, we measured the mRNA levels of several inflammatory markers, IL-4, IL-6, IL-8, IL-10, iNOS, and TNF-α in rat lung tissues using RT-PCR. As shown in Fig. 3A, rats exposed to oral Pb 100 mg/kg b.w. for 3 days presented significant increases in the mRNA expression levels of IL-4, IL-10, iNOS, TNF-α, which were associated with a significant decrease in IL-6 and IL-8 mRNA levels as compared to controls. To explore whether the effect of Pb on metabolizing enzymes is responsible for the detoxification of environmental pollutants, we measured the mRNA levels of CYP1A1, the downstream target for AhR and EphX1. Figure 3B shows that Pb increased the expression of CYP1A1 by approximately 50%, whereas downregulated EphX1 mRNA levels by 75% as compared to control.

### Effect of Pb exposure on serum inflammatory biomarkers levels

To determine the possible effects of Pb exposure on the levels of inflammatory biomarkers in the blood, we measured the capacity of high oral dose of Pb (100 mg/kg b.w.) to induce changes in the serum levels of immunological markers, such as MPO, PR-3, CK-MB, immunoglobulins, and LDH using ELISA. Table 2 illustrates that rats exposed to oral Pb 100 mg/kg b.w, expressed significant increases in the serum levels of CK-MB, LDH, and MPO by approximately 2-, 2.4-, and 1.5-fold, respectively. On the other hand, a significant decrease (40%) in immunoglobulin IgG was observed.

**Table 2:** Serum biomarkers analysis in rats exposed to once-daily dose of Pb (100 mg/kg, p.o.) for 3 days

Biomarkers	Control	Pb-treated
CK-MB (U/ml)	603.33±43.75	1196.37±134.83*
LDH (U/ml)	422±80.74	998.66±182.95*
Creatinine (μmol/l)	18±0.7	20.4±1.17
Urea (mmol/l)	6.55±0.02	7.06±0.25
MPO (U/ml)	0.55±0.06	0.84±0.05*
PR-3 (U/ml)	2.38±0.02	2.51±0.04
IgM (g/l)	0.45±0.01	0.39±0.04
IgG (g/l)	2.52±0.01	1.8±0.03*

Data are expressed as mean±SEM, *P<0.05 compared to the control (Pb 0 mg/kg b.w.) (Student *t*-test)

### Effect of Pb exposure on the profile of inorganic and organic compounds in rat lung tissues

GC-MS and ICP-MS were used to identify the organic and inorganic profiles, respectively. Table 3 shows the most important changes in the organic compound profile of rat lung tissues in response to oral Pb exposure (100 mg/kg b.w.) compared to those of a matched control. The organic compound profile of lung tissue homogenates reflects changes in inflammatory and oxidative stress-related metabolites in the Pb-exposed group. Interestingly, tyrosine p-octyl acetophenone fatty acid esters were only present in the lung tissue of Pb-exposed rats. Furthermore, GC-MS analysis revealed that the percentages of peak areas of 1-hexadecanol, 1-nonadecene, methyl stearate, and 1,2-octadecanediol were higher in Pb-treated group than control group (Table 3).

**Table 3:** The most important organic profiles identified by GC-MS analysis in lung homogenate of rats exposed to once-daily dose of Pb (100 mg/kg, p.o.) for 3 days

Compound name	MF	MW	RT (min)	PA%	
				Control	Pb-treated
3-Nitrotyrosine	C9H11NO3	181	7.26	0	5.34
p-Octylacetophenone	C16H24O	232	9.19	0	2.67
1-Hexadecanol	C16H34O	242	10.34	2.13	24.4
1-Nonadecene	C19H38	266	11.7	3.29	52.85
Methyl stearate	C19H38O2	298	12.55	4.85	26.25
1,2-Octadecanediol	C18H38O2	286	14.08	4.92	68.79

*MF*, molecular formula; *MW*, molecular weight; *RT*, retention time; *PA%*, peak area percentage. Note: all compounds were revealed with library similarity index (SI) scores above 90%

Table 4 shows the most important changes in the inorganic ion profile of rat lung tissues in response to oral Pb exposure (100 mg/kg b.w.) compared to those of a matched control. Importantly, the levels of magnesium and copper ions in lung homogenates of Pb-treated rats significantly decreased by approximately 400% and 7%, respectively, whereas selenium ion showed a 13% increase as compared to control groups.

**Table 4:** The most important inorganic profiles identified by using ICP-MS analysis in lung homogenate of rats exposed to once-daily dose of Pb (100 mg/kg, p.o.) for 3 days

Groups	Element levels (μg/L)	
	Control	Pb-treated
Magnesium (Mg)	18.33±0.02	3.63±0.01*
Copper (Cu)	3.86±0.001	3.59±0.06*
Selenium (Se)	1.41±0.003	1.6±0.02*

Data are expressed as mean±SEM, *P<0.05 compared to the control (Pb 0 mg/kg b.w.) (Student *t*-test)

### Effect of Pb exposure on NF-κB and AhR function in lung tissues

**Fig. 4:**
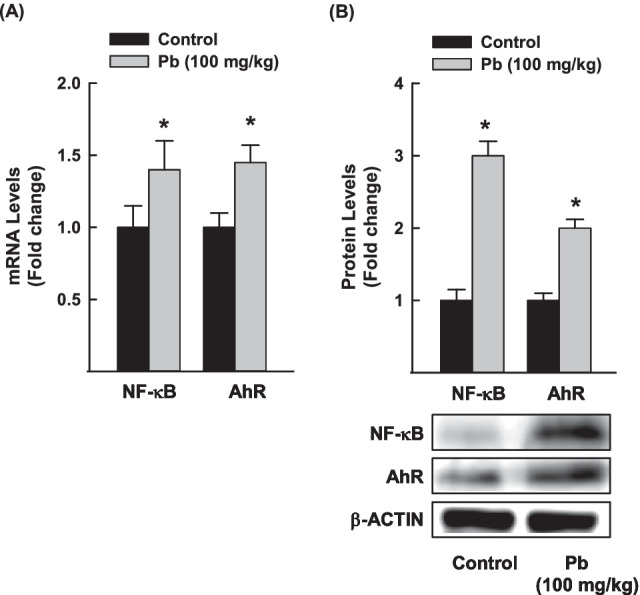
Effects of Pb exposure on the expression of NF-κB and AhR in rat lung tissues. NF-κB and AhR mRNA **A** and protein **B** levels were quantified using RT-PCR and Western blot analyses, respectively, normalized to the β-actin housekeeping gene. The values are presented as mean ± SEM (*n* = 3, duplicate). *p < 0.05 compared to the control (Pb 0 mg/kg b.w.)

To answer the question of whether NF-κB and AhR could have a role in Pb-induced lung inflammation in rats, we measured the effect of Pb on the mRNA and protein expressions of NF-κB and AhR in rat lung tissues. Figure 4 shows that treatment with Pb 100 mg/kg b.w. for 3 days resulted in a significant increase in NF-κB and AhR mRNA by approximately 45% (Fig. 4A). At the protein expression level, Pb significantly induced the protein expression of NF-κB and AhR by 3- and 2-fold, respectively (Fig. 4B), in a manner similar to mRNA.

## Discussion

In this study, we investigated (a) the distribution of Pb in rat lung tissues in comparison with other rat vital organs (heart and brain) and its impact on organs histology, (b) the effect of Pb on inflammatory and apoptotic markers in rat lung tissues and sera, and (c) the effect of Pb on the expression of NF-κB and AhR pathways in rat lung tissues. The key findings of the current work are that Pb after oral administration for three days highly distributed to the lung and induced inflammatory and apoptotic changes mediated through the activation of NF-κB and AhR.

Exposure to environmental pollutants is known to induce lung inflammation through the stimulation of several inflammatory mediators such as cytokines and interleukins (Moldoveanu et al. 2009). It is becoming increasingly clear that Pb-induced inflammation plays an important role in lung toxicity (Ansari et al. 2013; Korashy and El-Kadi 2008b; Wang et al. 2017; Zajda et al. 2017). Initially, in this study, we tested the distribution profile of Pb after oral administration to specifically determine which organ is most targeted by Pb. ICP-MS analysis shows that Pb was mainly accumulated in the lungs followed by the heart and the brain. The results are consistent with previously reported studies that demonstrated that the lung is one of the largest soft tissues for Pb absorption and accumulation (Peter and Strunc 1983; Sun et al. 2009). The highest distribution of Pb to the lung was associated with clinical manifestations of toxicity. First, changes in lung histology were characterized by the presence of severe interstitial inflammation and fibrosis in the lung tissues after 3 days of administration of Pb 100 mg/kg b.w., in a manner consistent with the findings of previous studies (Ansari et al. 2013; Chibowska et al. 2016). Second, a significant increase in the serum levels of several inflammatory markers such as LDH, CK-MB, and antibodies against neutrophil cytoplasmic antigens (MPO and PR-3 levels) and the decrease in IgG and IgM levels, suggesting the ability of Pb to induce lung and systemic inflammation. Although CK-MB and LDH are found in almost all tissues of the body and can be elevated in many pathological conditions, several previous studies have observed elevated serum levels of LDH and CK-MB in patients with chronic cough and lung inflammation (Faruqi et al. 2012) and lung cancer (Lee et al. 1985).

Three days of exposure to Pb induced severe lung inflammation as evidenced by (a) increased the gene expression of several inflammatory cytokines and mediators such as IL-4, IL-10, iNOS, TNF-α, and TGF-α and (b) inhibited the gene expression of anti-inflammatory markers such as IL-6 and IL-8. The release of IL-4 found to be linked with lung inflammatory diseases that are accompanied by NF-κB activation (Rokudai et al. 2006). Although IL-10 has been identified as an anti-inflammatory cytokine, its deficiency may be responsible for the activation of NF-κB and excessive inflammation. The IL-10 over expression in the lung of transgenic mice causes mucus metaplasia, tissue inflammation, and airway fibrosis (Lee et al. 2002; Saadane et al. 2005). What is supporting our findings is the observations of Tyagi and co-workers who reported that induction of macrophage-derived cytokines (TNF-α) and iNOS is associated with lung inflammation, and an increased risk of developing lung fibrosis and cancer (Beamer and Shepherd 2013; Tyagi et al. 2012). Both TNF-α and iNOS are considered inflammatory and apoptotic regulators (Nakazawa et al. 2017; Wang et al. 2018a). Recently, TNF-α and iNOS expressions have been associated with the activation of p53 and NF-κB, resulting in inflammation and apoptosis (Natarajan et al. 2018; Sawada et al. 2004).

Apoptosis is an important contributor to the pathophysiology of lung diseases. The involvement of apoptosis in Pb-induced lung inflammation is evidenced by (a) increasing the percentage of apoptotic cells in rat lungs with a proportional decrease in the percentage of healthy cells, (b) decreasing the expression of the anti-apoptotic BcL2, and (c) increasing TGF-α mRNA levels. Both BcL2 and TGF-α are involved in the modulation of apoptosis (Cory et al. 2003; Piacentini et al. 1991). Although physiological apoptosis does not induce inflammation, cell death and damage induced by chemical toxins might lead to leakage of cell contents into the adjacent tissues, causing the accumulation of neutrophils and the release of enzymes and oxygen radicals which enhance the inflammatory reaction (Haanen and Vermes 1995).

Perhaps the most interesting part of this study was the identification of potential organic and inorganic compounds that could medicate the inflammatory and apoptotic effects of Pb. GC-MS analysis of rat lung tissues has revealed an elevation of several organic compounds such as 1-hexadecanol, 1-nonadecene, methyl stearate 1,2-octadecanediol, and nitrotyrosine and p-octyl suggesting their role in subsequent inflammation (Pennathur et al. 2016; Sala et al. 2003; Sala et al. 2001). In this context, tyrosine compounds are known to play important roles in metabolism and have been shown to convert to nitrotyrosine upon nitration via peroxynitrite or/and MPO, which contributes to lung inflammatory diseases and apoptosis (Estévez et al. 1998; Haddad et al. 1994; Masuda et al. 2000; Moulian et al. 2001; Sheffield et al. 2006; Shin et al. 1996). The presence of nitrotyrosine in the lung tissue of Pb-exposed rats suggests the presence of reactive nitrogen molecules (peroxynitrite) and oxidative stress regulator (MPO), which mediates oxidative stress and inflammation (Jin et al. 2011; Wang et al. 2018b). However, analysis of the inorganic compound profile revealed that magnesium levels in rat lung homogenates decreased in response to Pb, indicating the protective role of magnesium in both the immune and respiratory systems (Mathew and Altura 1988). In this context, it has been demonstrated that magnesium deficiency promotes elastin degradation and excess intracellular calcium entry, leading to oxidation stress, nitration stress, and lung inflammation and dysfunction (Janssen 2017; Rayssiguier et al. 2010). Our results are in agreement with previous studies that showed that lung cancer patients exhibit lower serum levels of magnesium with higher Pb levels (Cobanoglu et al. 2010). In addition, sub-chronic Pb intoxication in rats causes a reduction in the tissues content of magnesium (Todorovic et al. 2008). Furthermore, it has been reported that magnesium could antagonize Pb-induced lung adenomas in mice through inhibiting Pb uptake by human amniotic epithelial cells (Guiet-Bara et al. 1990; Poirier et al. 1984).

Another marker that mediates Pb-induced lung inflammation and toxicity is EphX which plays a role in the protection against oxidative stress through the detoxification of toxic epoxide intermediates (Chen et al. 2011; Liu et al. 2013; Morisseau 2013). Thus, downregulation of EphX expression in response to Pb could be one of the mechanisms of Pb-induced lung inflammation (Chen et al. 2011, Liu et al. 2013, Morisseau 2013). Taking these observations with the ability of Pb to modulate the expression of xenobiotic-metabolizing enzymes such as CYP1A1 raises the question of the possible involvement of CYP1A1 in Pb-induced lung toxicity. To test this possibility, we measured the expression of CYP1A1 in Pb-exposed lung tissues. Interestingly, we found an inverse proportional increase in CYP1A1 and AhR mRNA levels with EphX1 levels, indicating the role of AhR/CYP1A1 in Pb-induced lung inflammation. It is becoming increasingly clear that the cross-talk between AhR and NF-κB signaling plays an important role in lung toxicity (Ansari et al. 2013; Korashy and El-Kadi 2008b; Wang et al. 2017; Zajda et al. 2017). Importantly, we found that activation of AhR/CYP1A1 in response to Pb was associated with a proportional increase in NF-κB mRNA in the lung tissues of the Pb-exposed group compared with the control. These results are in agreement with previous reports that demonstrated that Pb induced the activation of NF-κB and AhR in vitro and in vivo heart models (Ansari et al. 2013; Korashy and El-Kadi 2008a, b).

In conclusion, our results demonstrate that the lungs were the most vulnerable to the toxic effects of Pb at the molecular and cellular levels through the activation of inflammation and apoptosis, which are mediated via the modulation of AhR and NF-κB pathways. The induction of gene expressions of TNF-α, iNOS, and CYP1A1, while the reduction in the EphX gene, accumulation of lung fatty acids and 3-nitrotyrosine, reduction of lung tissues content of magnesium, supports the roles of oxidation stress, nitration stress, apoptosis, and inflammation in Pb-induced lung immunotoxicity.
