# Correction to: Lead nitrate induces inflammation and apoptosis in rat lungs through the activation of NF‑κB and AhR signaling pathways

**DOI:** 10.1007/s11356-022-20901-y

**Published:** 2022-05-21

**Authors:** Ibraheem M. Attafi, Saleh A. Bakheet, Sheikh F. Ahmad, Osamah M. Belali, Fawaz E. Alanazi, Suliman A. Aljarboa, Ibrahim A. AL‑Alallah, Hesham M. Korashy

**Affiliations:** 1 Department of Pharmacology & Toxicology, College of Pharmacy, King Saud University, Riyadh, Saudi Arabia; 2 Poison Control and Medical Forensic Chemistry Center, Jazan Health Affairs, Jazan, Saudi Arabia; 3 Aseer Central Hospital, Asser Health Affairs, Ministry of Health, Abha, Saudi Arabia; 4 Security Forces Hospital Program, Riyadh, Saudi Arabia; 5 Central Laboratory, Research Center, College of Pharmacy, King Saud University, Riyadh, Saudi Arabia; 6 Pathology and Clinical Laboratories Medicine, King Fahad Medical City, Riyadh, Saudi Arabia; 7 Department of Pharmaceutical Sciences, College of Pharmacy, QU Health, Qatar University, Doha, Qatar

**Correction to: Environmental Science and Pollution Research**

10.1007/s11356-022-19980-8

The sizing of images Figs. 3 and 4 is modified in the original published proof.

The Original article has been corrected.

